# Supplementary material for: Gene Silencing through CRISPR Interference in Mycoplasmas
Source: Microorganisms. 2022 Jun 5;10(6):1159. doi: 10.3390/microorganisms10061159 (PMC9229473; doi:10.3390/microorganisms10061159)
Supplement: Supplementary file 1 [file microorganisms-10-01159-s001.zip › figure S1-S5.pdf]

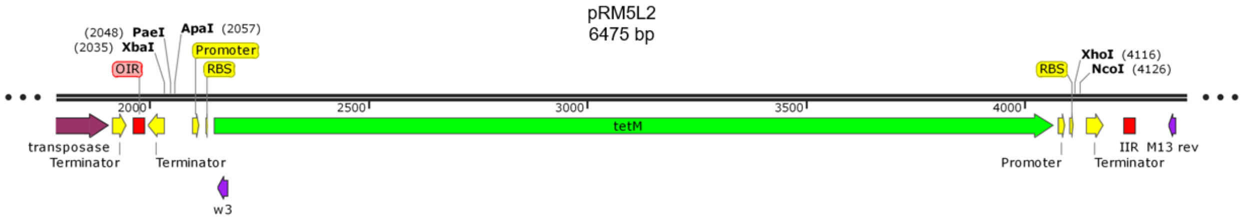

Fig. S1 pRLM5L2 transposon vector scheme. Promoter, RBS and terminator are yellow. OIR and IIR - inverted repeats for integration transposon into genome. W3 – primer, that is complementary to 5'-end of coding strand *tetM*, used for Sanger sequencing and validation position of transposon insertion.

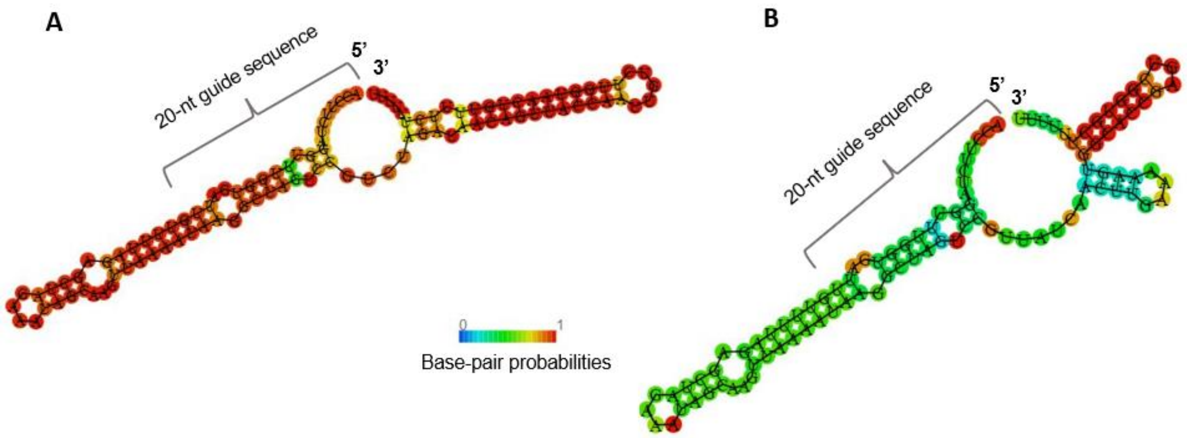

Fig. S2 Predicted secondary structure of sgRNAs. (A) sgRNA variant 1for *fur* knockdown with terminator sequence provided in pRM5L2 (located downstream of ApaI and XbaI sites). Guide sequence and gRNA exhibit a significant level of base pair interactions, compromising the formation of essential stem-loop structures **(B)** sgRNA variant 1 for *fur* knockdown with canonical *S. pyogenes* terminator. Guide sequence and gRNA exhibit weak interactions only. The color scale displays the base-pairing probability.

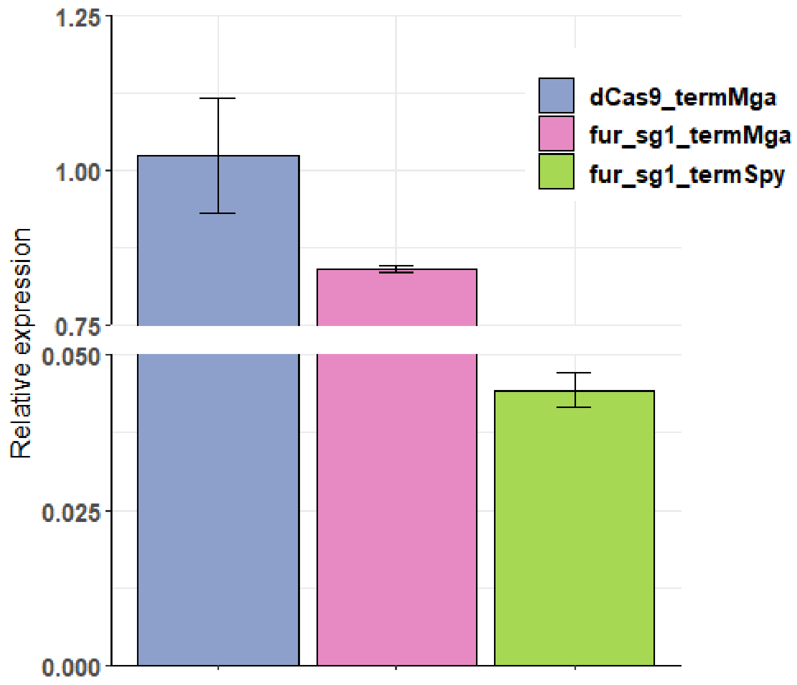

Fig. S3 Effect of sgRNA terminator sequence on *fur* repression. Quantitative RT-PCR analysis showing levels of *fur* in *dcas9* and *fur*-sgRNA1 containing AT-rich terminator (fur_sg1_termMga)-expressing transformants or Spy terminator sequence (fur_sg1_termSpy) compared to those in wild-type bacteria. dCas9 – transformants with expression only *dcas9*.

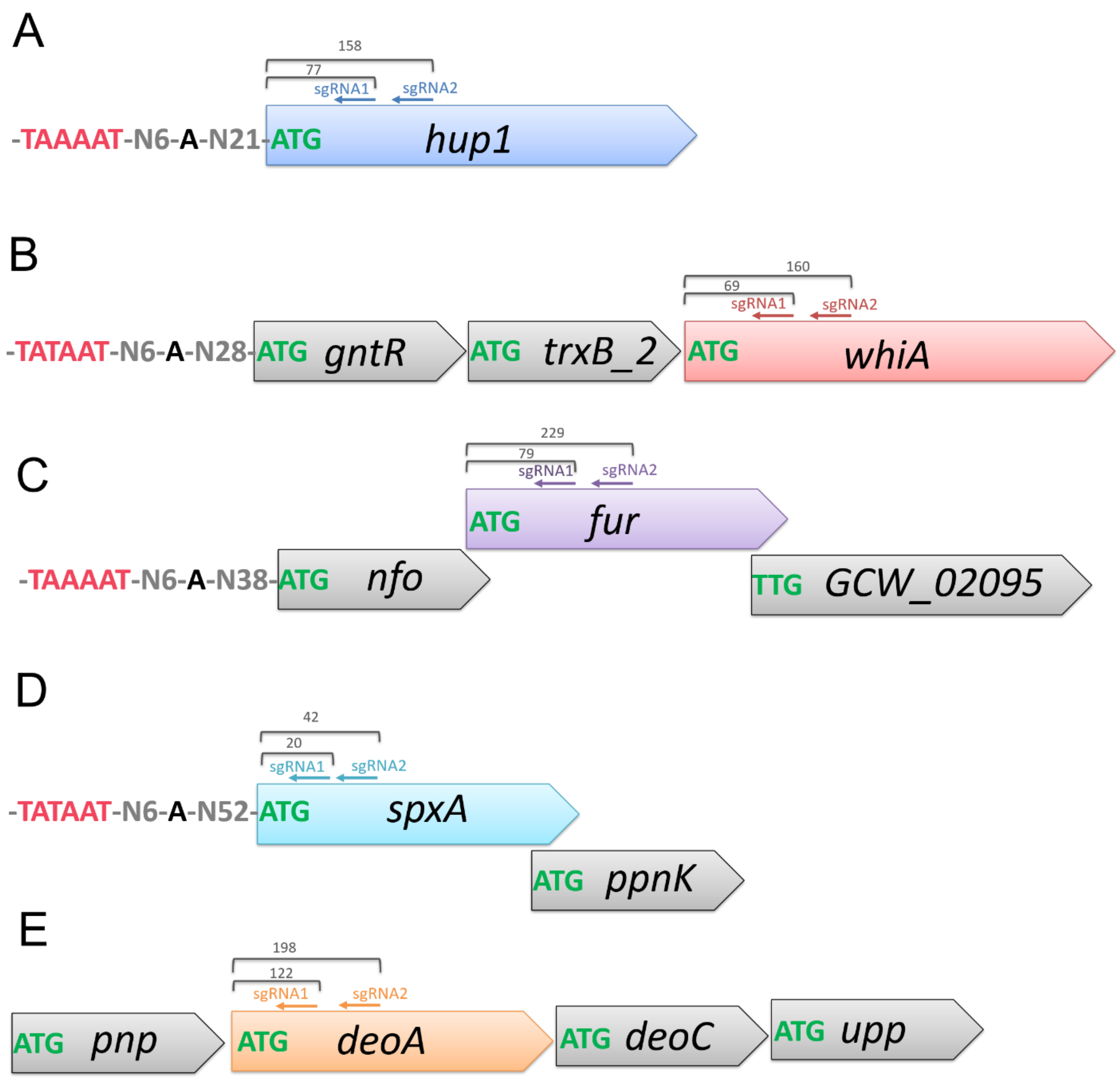

Fig. S4 Structure of the operons, contained genes of interest. Arrows indicate the position of 20-nt target sequences of designed sgRNAs; -10 box of promoter is red, initiator nucleotide is black, start codon is green. **(A)-(D)** *M. gallisepticum* targets, **(E)** *M. hominis* target. Our previous data of whole-genome mapping of transcription start sites for *M. gallisepticum* was used [Mazin P V., Fisunov GY, Gorbachev AY, Kapitskaya KY, Altukhov IA, Semashko TA, et al. Transcriptome analysis reveals novel regulatory mechanisms in a genome-reduced bacterium. Nucleic Acids Res. 2014;42(21):13254–68.].

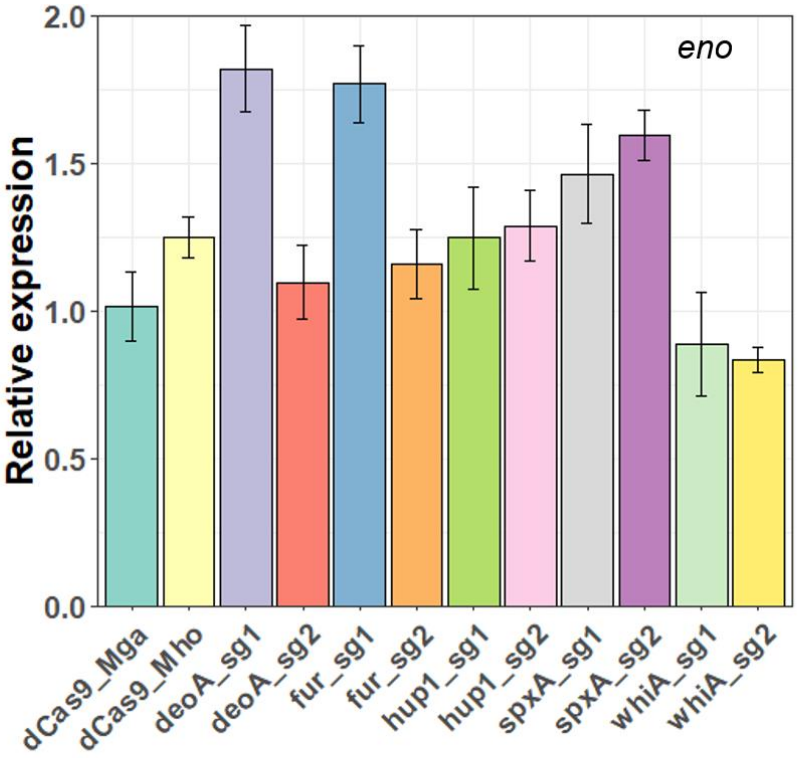

Fig. S5 Enolase mRNA expression level in all transformants. As a control, expression level of housekeeping *eno* in transformants relative of it in wild-type *M. gallisepticum* or *M. hominis* for dCas9-Mho, deoA_sg1 and deoA_sg2 transformants was measured.
